# Altered high-energy phosphate and membrane metabolism in Pelizaeus–Merzbacher disease using phosphorus magnetic resonance spectroscopy

**DOI:** 10.1093/braincomms/fcac202

**Published:** 2022-08-05

**Authors:** Jeremy J Laukka, Kevin M Kain, Anirudha S Rathnam, Jasloveleen Sohi, Dalal Khatib, John Kamholz, Jeffrey A Stanley

**Affiliations:** Department of Medical Education, University of Toledo College of Medicine and Life Sciences, Toledo, OH, USA; Department of Neurology, University of Toledo College of Medicine and Life Sciences, Toledo, OH, USA; College of Osteopathic Medicine, Kansas City University, Kansas City, MO, USA; Department of Neurology, Henry Ford Health System, Detroit, MI, USA; Department of Neurology, Center for Molecular Medicine and Genetics, Wayne State University School of Medicine, MI, USA; Department of Psychiatry and Behavioral Neurosciences, Wayne State University School of Medicine, Detroit, MI, USA; Department of Neurology, Center for Molecular Medicine and Genetics, Wayne State University School of Medicine, MI, USA; Department of Neurology, University of Iowa Carver College of Medicine, Iowa City, IA, USA; Department of Psychiatry and Behavioral Neurosciences, Wayne State University School of Medicine, Detroit, MI, USA

**Keywords:** Pelizaeus–Merzbacher disease, phosphorus magnetic resonance spectroscopy, biochemistry, high-energy phosphate, membrane phospholipids

## Abstract

Pelizaeus–Merzbacher disease is an X-linked recessive leucodystrophy of the central nervous system caused by mutations affecting the major myelin protein, proteolipid protein 1. The extent of the altered *in vivo* neurochemistry of protein, proteolipid protein 1 duplications, the most common form of Pelizaeus–Merzbacher disease, is, however, poorly understood. Phosphorus magnetic resonance spectroscopy is the only *in vivo* technique that can assess the biochemistry associated with high-energy phosphate and membrane phospholipid metabolism across different cortical, subcortical and white matter areas. In this cross-sectional study, whole-brain, multi-voxel phosphorus magnetic resonance spectroscopy was acquired at 3 T on 14 patients with Pelizaeus–Merzbacher disease with protein, proteolipid protein 1 duplications and 23 healthy controls (all males). Anabolic and catabolic levels of membrane phospholipids (phosphocholine and phosphoethanolamine, and glycerophosphoethanolamine and glycerophosphocholine, respectively), as well as phosphocreatine, inorganic orthophosphate and adenosine triphosphate levels relative to the total phosphorus magnetic resonance spectroscopy signal from 12 different cortical and subcortical areas were compared between the two groups. Independent of brain area, phosphocholine, glycerophosphoethanolamine and inorganic orthophosphate levels were significantly lower (*P* = 0.0025, *P* < 0.0001 and *P* = 0.0002) and phosphocreatine levels were significantly higher (*P* < 0.0001) in Pelizaeus–Merzbacher disease patients compared with controls. Additionally, there was a significant group-by-brain area interaction for phosphocreatine with *post-hoc* analyses demonstrating significantly higher phosphocreatine levels in patients with Pelizaeus–Merzbacher disease compared with controls across multiple brain areas (anterior and posterior white matter, superior parietal lobe, posterior cingulate cortex, hippocampus, occipital cortex, striatum and thalamus; all *P* ≤ 0.0042). Phosphoethanolamine, glycerophosphoethanolamine and adenosine triphosphate levels were not significantly different between groups. For the first-time, widespread alterations in phosphorus magnetic resonance spectroscopy metabolite levels of Pelizaeus–Merzbacher disease patients are being reported. Specifically, increased high-energy phosphate storage levels of phosphocreatine concomitant with decreased inorganic orthophosphate across multiple areas suggest a widespread reduction in the high-energy phosphate utilization in Pelizaeus–Merzbacher disease, and the membrane phospholipid metabolite deficits suggest a widespread degradation in the neuropil content/maintenance of patients with Pelizaeus–Merzbacher disease which includes axons, dendrites and astrocytes within cortex and the myelin microstructure and oligodendrocytes within white matter. These results provide greater insight into the neuropathology of Pelizaeus–Merzbacher disease both in terms of energy expenditure and membrane phospholipid metabolites. Future longitudinal studies are warranted to investigate the utility of phosphorus magnetic resonance spectroscopy as surrogate biomarkers in monitoring treatment intervention for Pelizaeus–Merzbacher disease.

## Teaser

The extent of the altered *in vivo* neurochemistry in Pelizaeus–Merzbacher disease, an X-linked recessive leucodystrophy, is poorly understood. In this study, Laukka *et al*. report widespread biochemical alterations in patients with Pelizaeus–Merzbacher disease compared with controls utilizing phosphorous magnetic resonance spectroscopy. Results implicate energy expenditure and membrane phospholipid metabolism in Pelizaeus–Merzbacher disease.

## Graphical Abstract

**Graphical abstract:**
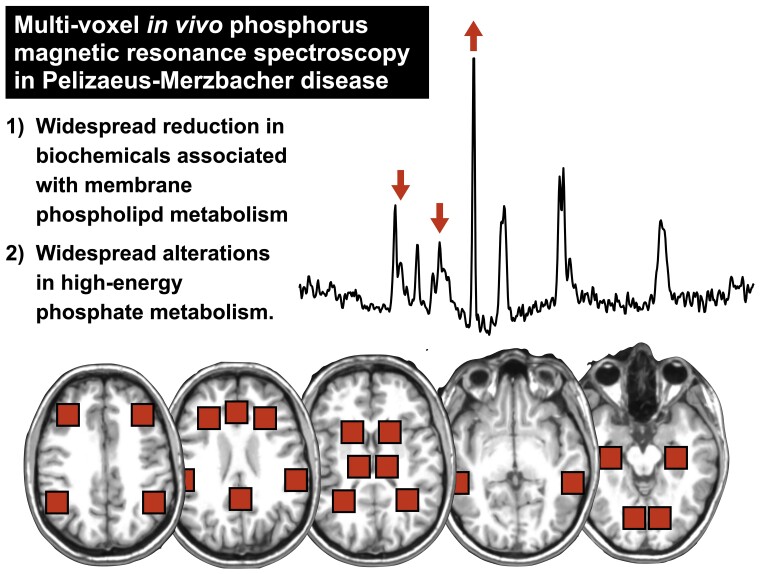

## Introduction

Pelizaeus–Merzbacher disease is an X-linked leucodystrophy caused by mutations in the gene encoding of the major CNS myelin structural protein, proteolipid protein 1 (PLP1). The most common cause of Pelizaeus–Merzbacher disease is through the duplication of the X-chromosome that encodes the PLP1 gene, which accounts for 50 to 75% of cases.^1^ Pelizaeus–Merzbacher disease is also caused by a variety of point mutations in the PLP1 gene, altering the PLP1 structure and expression. Overexpression of PLP1 causes diffuse demyelination, axonal loss and infiltration of myelin with both macrophages and microglia, but does not alter normal myelin development.^2^ The cellular and molecular mechanisms causing demyelination in patients with PLP1 duplications are not well understood but may include decreased energy utilization.^3,4^

Structural T_1_- and T_2_-weighted MRI studies have demonstrated distinct differences in the signal intensity of white matter (WM) areas in patients with Pelizaeus–Merzbacher disease^5,6^ with a variety of PLP1 mutations, suggesting hypomyelination, dysmyelination and demyelination due to alteration in both oligodendrocyte function and PLP1 function and expression.^7^ Structural MRI studies have also demonstrated volume reductions in frontal WM, arcuate fibres and the internal capsule,^8^ as well as diffuse atrophy throughout the brain of patients with Pelizaeus–Merzbacher disease^9^ although with similar proportion of grey matter (GM) to WM compared with healthy individuals.^8^ Lastly, diffusion tensor imaging studies have also implicated primarily WM areas in patients with Pelizaeus–Merzbacher disease with some alterations in GM areas.^10^ In all, the evidence provides the extent of the known areas of brain pathology in patients with Pelizaeus–Merzbacher disease but provide little insight into the underlying cellular and molecular pathophysiology of the effects of PLP1 duplications.

Hüttemann *et al*.^3^ and Appikatla *et al*.^4^ have demonstrated in transgenic mice that overexpress PLP1, that PLP1 can be inserted into the mitochondrial membrane as well as into myelin. Transgenic mice with increased PLP1 gene dosage have markedly decreased concentrations of brain adenosine triphosphate (ATP) and altered expression of cytochrome oxidase, Complex IV of the mitochondrial respiratory chain. In addition, tissue culture cells transfected with wild-type PLP1 acidify their culture media, also suggesting that mitochondrial function has been altered. Together, this suggests that mitochondrial dysfunction impacting energy utilization may play a role in the pathogenesis of Pelizaeus–Merzbacher disease.

**Figure 1:**
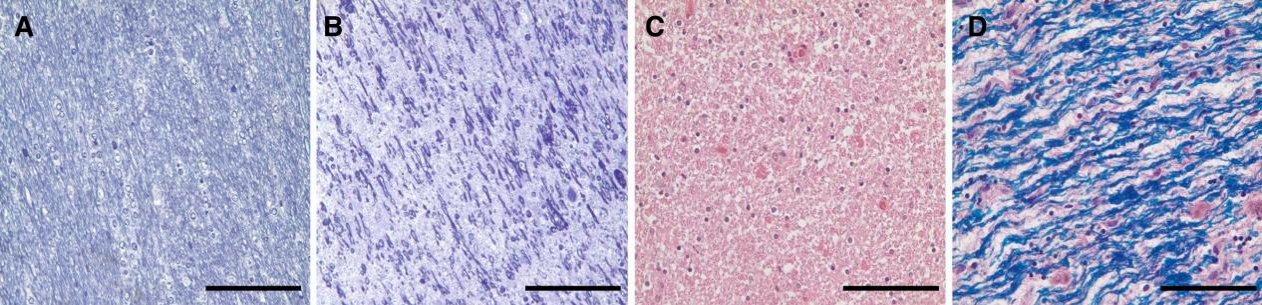
**Pathological analysis of WM from a 60-year-old patient with PLP1 duplication.** (**A**) Toluidine blue staining of a normal control subject. (**B**) Toluidine blue staining of patient with Pelizaeus–Merzbacher disease showing thinning of myelin and reduced axonal fibre density with an increase composition of myelin degradation products. (**C**) H&E staining also demonstrates very thin myelin and reduced numbers of oligodendrocytes. There are also irregular non-uniform aggregates which are presumably degradation products of myelin. (**D**) HE-LFB staining shows a reduction and thinning of myelinated fibres with diffuse myelin degradation products. Scale 50 µm.

Phosphorus magnetic resonance spectroscopy (³¹P MRS) is the only non-invasive method that can assess the neurobiological basis related to high-energy phosphate [ATP and the high-energy phosphate store, phosphocreatine (PCr) and inorganic orthophosphate (Pi)] and membrane phospholipid (MPL) metabolites [precursors, phosphocholine (PC) and phosphoethanolamine (PE), and breakdown products, glycerophosphocholine (GPC) and glycerophosphoethanolamine (GPE)]. The latter reflects the molecular biochemistry and cellular density of axons, dendrites, astrocytes (neuropil).^11–14^ Moreover, both PC and PE act as both anabolic and catabolic products of sphingomyelin and hence, reflect the metabolism of oligodendrocytes and myelin.^15^ Stained sections from the brain of a patient with Pelizaeus–Merzbacher disease with PLP1 duplication in Fig. 1 demonstrate the cellular substrates analysed with ³¹P MRS study—decreased density of axons, thinner myelin and increase of myelin breakdown products.

To advance our understanding of the cellular and molecular biochemistry of this disease, *in vivo* ³¹P MRS was applied to investigate regional differences in high-energy phosphate metabolites and in MPL precursor and breakdown products between patients with Pelizaeus–Merzbacher disease with PLP1 gene duplications and healthy individuals. We hypothesized widespread differences in the ³¹P MRS biochemistry between both groups that would implicate the high-energy phosphate utilization and the cellular density of both WM and GM in the pathology of Pelizaeus–Merzbacher disease.

## Materials and methods

### Participants

The 14 patients with Pelizaeus–Merzbacher disease and PLP1 duplications (all males; mean age 18.3 ± 7.3 years; age range of 9.5–30.8 years) were recruited between 2012 and 2014 with the help of the Pelizaeus–Merzbacher Disease Foundation (http://pmdfoundation.org/?page_id=343). The 23 healthy individuals matched in age and sex (all males; mean age 16.1 ± 6.1 years; age range of 9.7–29.6 years) were recruited between 2010 and 2014 from Detroit and the Detroit metropolitan area. The upper and lower age range was selected to restrict the sample to adolescents and young adults and hence, minimize any age effects.

All healthy participants were screened via an interview and a written questionnaire regarding medical history and disqualified from participation if they had a history of neurological/psychiatric illness or head trauma. Consent obtained from all participants followed the procedures of the Wayne State University institutional review board. Parental consent was required from participants under the age of 18 years.

### Whole-brain, multi-voxel ³¹P MRS acquisition

The structural MRI and the ³¹P MRS data collection were conducted on a 3 T Siemens Verio whole-body system (Siemens, Germany) using a using a dual-tuned ³¹P/¹H transmit/receive volume head coil (Advanced Imaging Research, Inc., Cleveland, OH, USA). A three-plane MRI localizer was first collected followed by a set of sagittal and axial scout MRI images using the 2D fast spin-echo sequence, which were used to prescribe the ³¹P MRS measurement. The ³¹P MRS acquisition sequence included a single slab-selective excitation radiofrequency pulse followed by phase encoding pulses to spatially encode in all three directions. The slab in the axial plane was placed parallel to the AC-PC line and utilized to limit the localization within the brain. The experimental parameters were: 3D FID_CSI sequence (Siemens system) modified with a pre-acquisition delay of 1.43 ms, FOV = 340 × 340 × 170 mm^3^, slab thickness = 120 mm, acquired matrix = 14 × 14 × 8, zero-filled to 16 × 16 × 8, nominal voxel dimension = 2.125 × 2.125 × 2.125 cm^3^, repetition time TR = 0.54 s, spectral bandwidth = 3.3 kHz, 64 averages (weighted-average k-space), elliptical k-space sampling, with ¹H-decoupling and acquisition time 23 min. A set of T_1_-weighted MRI images was also collected during the ³¹P MRS session as well as with a single-tuned 32-channel ¹H volume head coil, which proceeded the ³¹P MRS. The T_1_-weighted MRI images (1 mm^3^ spatial resolution) collected with the dual-tuned head coil was used to co-register the subject space of the ³¹P MRS to the high-quality T_1_-weighted MRI images (1 mm^3^ spatial resolution) collected with the single-tuned ¹H volume head coil.

### Post-processing and quantification

**Figure 2:**
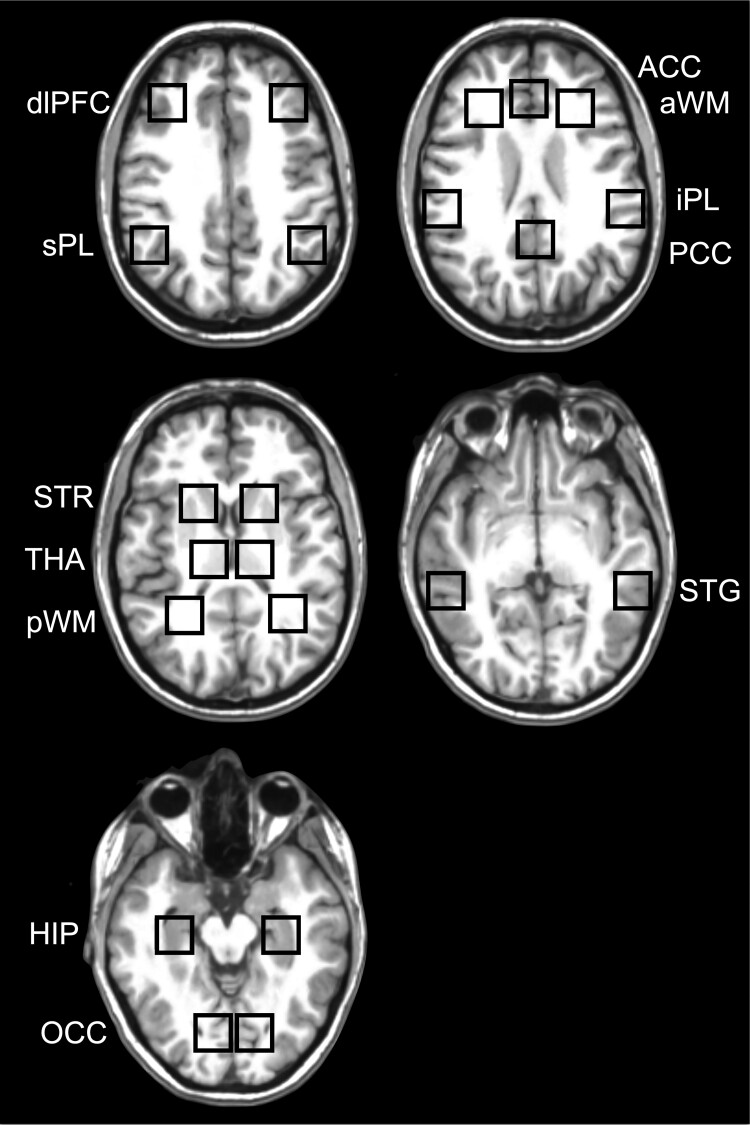
**Anatomical placement of the ³¹P MRS voxels.** Five different in-plane axial views of the 12 right/left and medial regions of interest as indicated by the black square outlines that include the anterior and posterior WM (aWM and pWM), dorsolateral prefrontal cortex (dlPFC), inferior and superior parietal lobe (iPL and sPL), superior temporal gyrus (STG), anterior/body of the hippocampus (HIP), occipital cortex (OCC), striatum (STR), thalamus (THA) and the medial anterior and posterior cingulate (ACC and PCC).

The post-processing included extracting and quantifying the ³¹P MRS signal from the different voxel locations. The procedure from start to finish was 100% automated (i.e. fully independent of operator input)^16^ using in-house software (C-shell UNIX scripts, MATLAB; The Math Works, Inc.) and FSL^17^ and FreeSurfer^18,19^ tools as well as the Marquardt^20^ non-linear time-domain MRS fitting programme (Fitman, Dr Rob Bartha, University of Western Ontario). Twelve different right and left voxel locations of interest were predefined anatomically on the MNI template brain and co-registered to the subject space by co-registering the T_1_-weighted images of the subject to the MNI template brain (12 degrees of freedom) and re-mapping the voxels to the subject space via the inverse transformation.^16^ The coordinates of each voxel location in subject space were then used to shift mathematically the 3D multi-voxel grid by applying a phase shift in the k-space to ensure the ³¹P MRS voxel was centred at each location and then the ³¹P signal at each location was extracted for quantification. This highly innovative procedure ensured that the voxel placements were consistently and systematically placed in the specified anatomical locations between subjects and has been demonstrated to be highly accurate and reliable.^16^ The voxel locations of interest included the right and left anterior and posterior WM (aWM and pWM), dorsolateral prefrontal cortex, inferior and superior parietal lobe (iPL and sPL), superior temporal gyrus (STG), anterior/body of the hippocampus (HIP), occipital cortex, striatum (STR) thalamus (THA) and the medial anterior and posterior cingulate (ACC and PCC). Figure 2 shows an example of the 12 voxel locations applied to each subject.

**Figure 3:**
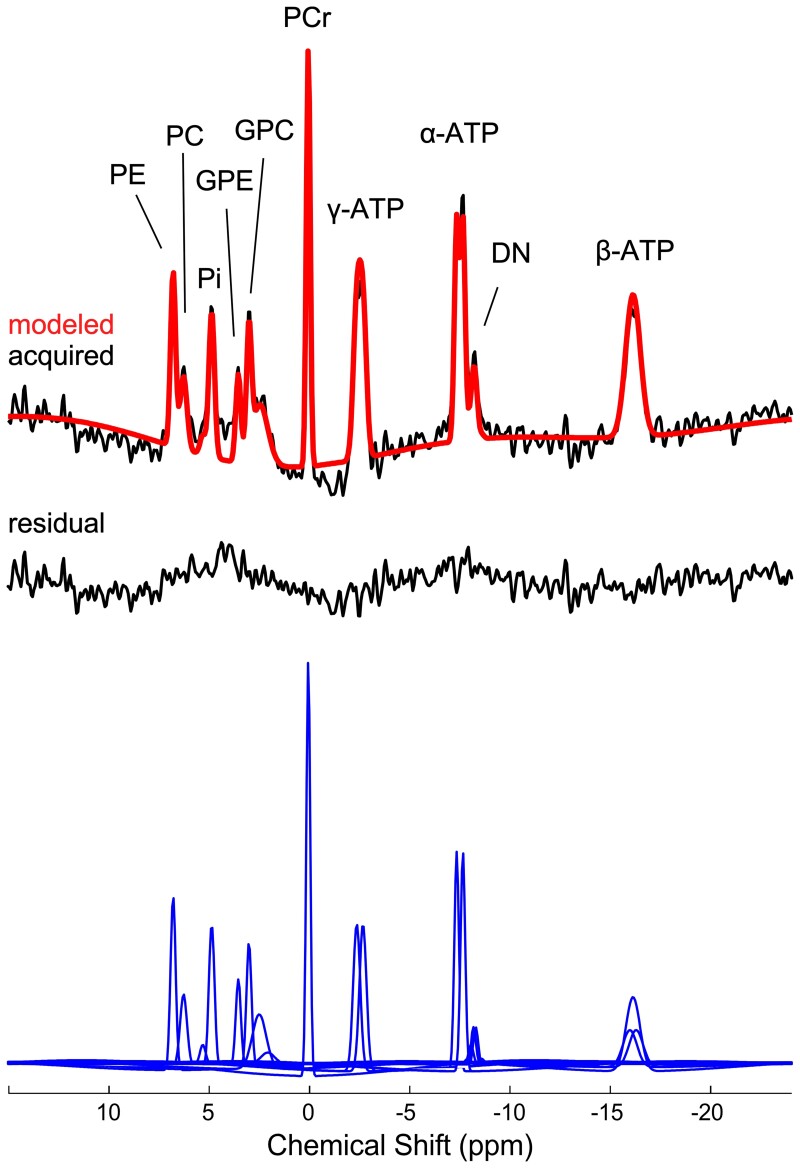
**Example of a quantified *in vivo* ³¹P MRS spectrum extracted from the left posterior WM area (pWM) of a participant.** The top includes the Fourier transformation of the acquired (black line) and modelled (red line) signal, and the residual (difference between acquired and modelled signal) is shown below (black line). The individual modelled spectral peaks are shown at the bottom (blue line) and the assignment of the spectral peaks are as indicated.

In the k-space domain of the ³¹P MRS data, a 75% Hamming window was applied, which resulted in estimated effective voxel size of approximately 15 cm^3^; an estimate that also accounted for the reduced k-space sampling during the acquisition. In the time domain of the ³¹P MRS dimension, a 5 Hz Gaussian line broadening was applied prior to quantification of the data. In quantifying the PE, PC, Pi, GPE, GPC, MPL, PCr, dinucleotides and ATP (two doublets and one triplet) resonances, the ³¹P MRS signal was modelled with 21 Gaussian damped sinusoids in the time domain using the appropriate a priori knowledge.^21^ PE, PC, GPE and GPC were modelled as singlets due to the applied ¹H-decoupling and the chemical shift of 2.23 p.p.m. for the MPL is consistent with prior reports of identifying a signal from less mobile phosphodiester moieties that have a relatively intermediate correlation time and are part of small MPL structures when the pre-acquisition delay time is relatively short (e.g. micelles, synaptic vesicles and transport/secretory vesicles associated with the Golgi and endoplasmic reticulum).^22–27^ ³¹P MRS spectra of poor spectral quality with spectral linewidth >30 Hz for PCr, or >50 Hz for PE, PC, GPC or GPE were excluded from the statistical analysis. In total, 35 out of 814 spectra (or 4% of the data) were excluded. The outcome metabolite measurements were expressed as mole% relative to the total ³¹P MRS signal, which have been demonstrated to provide equivalent results compared with expressing values as an absolute concentration.^28^ An example of a modelled spectrum from a key region of interest is shown in Fig. 3.

### Statistical analyses

Two regression analyses based on the generalized estimating equation methodology (PROC GENMOD; SAS Institute Inc.) were used, which included: (i) subject group (Pelizaeus–Merzbacher disease versus HC), age, region (12 voxel regions) and side (right and left) as the main effect terms to access the main group effect independent of region effects; and (ii) subject group, age, region, side and the group-by-region interaction as the main terms to access any group-by-region interactions. The dependent variables included PE, PC, GPE, GPC, PCr, Pi and ATP expressed as a mole% relative to the total ³¹P MRS signal from the 12 areas of interest, the right and left measurements across regions were treated as repeated measures in each analysis, and *P*-values of ≤0.0042 were considered significant for the *post-hoc* analyses in the second model reflecting the Bonferroni correction.

## Results

### Quality of the ³¹P MRS data

The ¹H-decoupling was effective in reducing the spectral broadening and subsequently allowed the delineation and quantification of the individual phosphomonoesters (PE and PC) and phosphodiesters (GPE and GPC), as demonstrated in Fig. 3. The spectral linewidths of PCr, Pi, ATP, PE, PC, GPE and GPC were not statically different between groups across all regions or within each region (Supplementary Table 2). The overall mean signal-to-noise ratio of PCr (±standard deviation) was 3.6 ± 0.7, which ranged from 3.1 ± 0.1 in the sPL to 4.2 ± 0.1 in the pWM. No data was rejected due to poor quality on the ³¹P MRS.

### Group effect across all 12 brain regions studied

**Figure 4:**
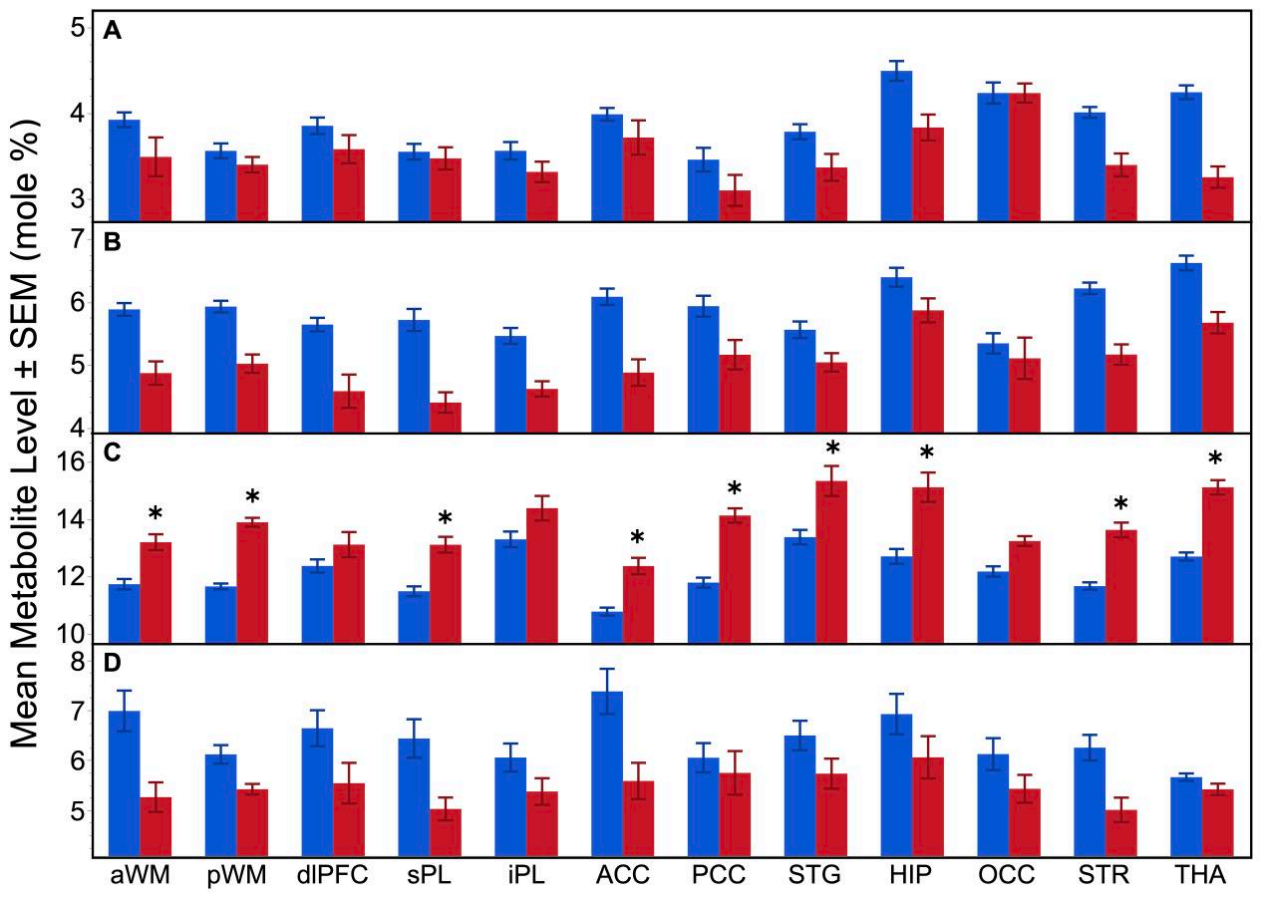
**³¹P MRS metabolite levels between groups and across regions.** Mean PC (**A**), GPC (**B**), PCr (**C**) and Pi (**D**) levels (±standard error of the mean) of the 12 regions of interest for the patients with Pelizaeus–Merzbacher disease (red bars) and healthy participants (blue bars). Significant group differences after applying the Bonferroni correction (*P* < 0.0042) from *post-hoc* analyses are indicated by the * symbol.

Patients with Pelizaeus–Merzbacher disease demonstrated a significant reduction in MPL precursor levels, PC (**χ^2^** = 9.17, *P* = 0.0025), MPL breakdown products, GPC (**χ^2^** = 18.3, *P* < 0.0001) and Pi (χ^2^ = 13.7; *P* = 0.0002) compared with healthy participants (Fig. 4A, B and D; Table 1). Moreover, there was a widespread increase in the high-energy store, PCr levels (**χ^2^** = 18.06, *P* < 0.0001) compared with healthy participants (Fig. 4C; Table 1). The group-by-region interaction was also significant for PCr (**χ^2^** = 21.93, *P* = 0.025; Table 1) with *post-hoc* analyses demonstrating higher PCr levels in the aWM, pWM, sPL, ACC, PCC, STG, HIP, STR and THA (Fig. 4C). The other metabolites, PE, GPE and ATP levels, were not significantly different between groups nor demonstrated regional effects (Table 1; Supplementary Table 1). Though, not part of the main objectives, these significant differences in the MPL and high-energy phosphate metabolites were observed across both adolescents and young adults based on the lack of any age effects (i.e. non-significant group-by-age interactions effects that were not reported), and were identical when expressing the metabolites as a ratio relative to ATP (Supplementary Tables 3 and 4).

**Table 1:** Results from the main group effect and group-by-region interaction for the metabolite levels

Metabolites	Main group effect	Main group-by-region interaction effect
PE	n.s. (χ^2^ = 0.00; *P* = 0.95)	n.s. (χ^2^ = 13.98; *P* = 0.23)
PC	↓ PMD versus controls (χ^2^ = 9.17; *P* = 0.0025)	n.s. (χ^2^ = 14.87; *P* = 0.19)
GPE	n.s. (χ^2^ = 2.04; *P* = 0.15)	n.s. (χ^2^ = 12.69; *P* = 0.31)
GPC	↓ PMD versus controls (χ^2^ = 18.3; *P* < 0.0001)	n.s. (χ^2^ = 13.89; *P* = 0.24)
PCr	↑ PMD versus controls (χ^2^ = 18.06; *P* < 0.0001)	χ^2^ = 21.93; *P* = 0.025. *Post-hoc* analyses of regions demonstrating significant increased PCr in PMD versus controls: aWM, pWM, sPL, ACC, PCC, STG, HIP, STR and THA (all *P* < 0.0042)
Pi	↓ PMD versus controls (χ^2^ = 13.7; *P* = 0.0002)	n.s. (χ^2^ = 12.9; *P* = 0.30)
ATP	n.s. (χ^2^ = 0.69; *P* = 0.41)	n.s. (χ^2^ = 11.63; *P* = 0.39)

n.s. = not significant; PMD, Pelizaeus–Merzbacher disease.

## Discussion

To our knowledge, this is the first study to report a widespread reduction in precursor levels and breakdown products of MPL as well as a widespread increase in high-energy store of PCr and reduction of Pi in patients with PLP1 duplications compared with healthy individuals. Specifically, PC, GPC and Pi levels were significantly reduced, and PCr levels were significantly increased in patients with Pelizaeus–Merzbacher disease across major anterior and posterior WM areas (Fig. 3) while ATP levels, which are well buffered, were not significantly different between groups. It is important to emphasize that the metabolite measurements were expressed as a mole% relative to the total ³¹P MRS signal and not as an absolute concentration level; however, there is evidence demonstrating equivalent results between expressing values as a mole% and absolute concentration.^28^ Additionally, the significant group differences were replicated when expressing the metabolite measurements as a ratio relative to ATP (Supplementary Tables 3 and 4). Collectively, this provides confidence that the mole% differences between groups reflect differences in metabolite levels.

### Deficits in MPL precursor and breakdown product levels in patients with Pelizaeus–Merzbacher disease

Although the spatial resolution of *in vivo* ³¹P MRS may be poorer compared with the more common ¹H MRS due to the lower sensitivity of the ³¹P nuclei, its specificity to the molecular biology and metabolic processes is much greater.^29,30^ In tissue, MPLs naturally form membrane bilayers that physically separate the intracellular components from the extracellular environment of different cell entities such as axons, dendrites, astrocytes (neuropil) as well as oligodendrocytes and myelin. During periods of neuropil development or myelination of WM tracts, *in vivo* human and *ex vivo* rat brain ³¹P MRS studies have consistently shown high MPL precursor levels of PE and PC reflecting the high demand of active MPL synthesis and metabolism of myelin.^11,15,29,30^ At the time and site of neuritic sprouting in an hippocampal lesion rat model, elevated MPL precursor levels, PC, concomitant with elevated MPL levels, phosphatidylcholine, have also been reported.^31^ Additionally, MPL are also constantly being turned over reflecting the ongoing reorganization/plasticity and maintenance of synaptic connections. Therefore, the observed deficits of MPL precursor levels and breakdown products, which were specific to PC and GPC, respectively, in patients with Pelizaeus–Merzbacher disease compared with healthy individuals suggests significant and widespread decrease both in axon density in cortex and in myelin in WM. This is consistent with evidence of decreased number of oligodendrocytes and related known processes of demyelination and axonal injury in patients with Pelizaeus–Merzbacher disease.^32^ In addition, there might also be metabolic abnormalities in oligodendrocyte function and myelin maintenance.

### Phosphocreatine levels in patients with Pelizaeus–Merzbacher disease

All cells native to the CNS, including neurons and glia, express creatine kinase (CK), the enzyme that catalyses the equilibrium among, PCr, adenosine diphosphate (ADP), creatine and ATP. Through CK equilibrium, PCr preserves a high-energy ratio of ATP/ADP by maintaining low ADP concentration. PCr is a high-energy compound that is used by the cell to maintain adequate ATP stores. PCr is synthesized in mitochondria by mitochondrial creatine phosphokinase, transported to the cytoplasm, and then used to regenerate ATP from ADP by the cytosolic creatine phosphokinase. Figure 4 shows a widespread increase in PCr levels and decrease in Pi levels across all brain regions analysed, which includes cortical and subcortical WM areas. Also, nine of which were statistically significant in the *post-hoc* analyses for PCr (*P* < 0.0042). Past ³¹P MRS studies have demonstrated the opposite of decreased PCr and increased Pi during processes reflecting increased high-energy utilization.^33^ In this study, increased PCr levels and decreased Pi levels concomitant with non-significant differences in ATP levels between groups suggest decreased energy utilization in the brains of patients with Pelizaeus–Merzbacher disease, possibly a result of decreased cytosolic CK activity^34^ as suggested by Hüttemann *et al*.^3^ and Appikatla *et al*.^4^.^3,4^ The lack of group differences in ATP in our study is probably a result of strong buffering of ATP pools. Interestingly, Zhang and co-workers^35^ also found increased levels of PCr in the brains of mouse models of Huntington disease, as well as the brain from an individual with this disease. In addition, they also found decreased levels of CK activity, both the brain-specific BB isoform and the mitochondrial isoform, suggesting that the decrease in CK activity may lead the accumulation of PCr that cannot utilize its high-energy phosphate. Taken together, our data from ³¹P MRS analysis of individuals with Pelizaeus–Merzbacher disease are consistent with the notion that a deficit in high-energy metabolism is a part of the pathophysiology of this disease. These data may serve as a way to identify potential surrogate biomarkers to follow treatment of patients in the future.

The number of pathologic studies focused on examining autopsy tissue from human patients with PLP1 duplications is limited.^36–38^ In contrast, there are *in vivo* ¹H MRS studies^39,40^ in patients with PLP1 duplications demonstrating increased *N*-acetyl-aspartate, PCr plus creatine (PCr + Cr) and myo-Inositol in WM but not in GM.^39,40^ ¹H MRS, however, lacks the biological specificity with respect to the interpretation of these results. For example, both PCr and Cr spectral peaks are indistinguishable on a ¹H MRS spectrum, and PCr and Cr are both reactants in the CK reaction and therefore, insensitive to reflect CK utilization. Likewise, GPC and PC are also indistinguishable and lack the specificity of implicating either the synthesis or degradation of MPL. Additionally, several studies in Pelizaeus–Merzbacher disease have reported ¹H MRS results expressing outcome measures as a ratio of metabolite levels, which further complicate the interpretation of results.^9,41–43^ Although these data are informative, ours is the first patient study using ^31^P MRS to quantify information on ATP and measure alterations in the bioenergetics in Pelizaeus–Merzbacher disease in a patient population with a uniform mutational aetiology.

### Limitations

There are limitations to this study including the following: (i) small sample size; (ii) age range of sample spanning from adolescents to young adulthood; (iii) lack of clinical data on symptom severity on the patients with Pelizaeus–Merzbacher disease for correlation analyses; (iv) relatively large voxel sizes leading to potential partial volume effects (i.e. heterogeneity in the portion of tissue types within voxels); (v) the process of shifting the CSI grid to ensure voxels were centred in the predefined anatomical locations only had 3 degrees of freedom (i.e. translation in the three orthogonal directions but no rotation or angulation), which may lead to subtle inaccuracies in the localization of the anatomical locations across participants; and (vi) expressing the biochemical levels as a mole% relative to the total ³¹P MRS signal and not as absolute concentration levels.^28^

## Conclusion

The ability to observe widespread biochemical differences in the brains of patients with Pelizaeus–Merzbacher disease and PLP1 duplications demonstrates that *in vivo* ³¹P MRS is not only useful to characterizing the biochemical differences *in vivo* associated with Pelizaeus–Merzbacher disease but could also be used as a tool for tracking treatment progress. Several new treatments are being targeted at restoring function to the genetically abnormal oligodendrocytes or using stem cells to transplant healthy oligodendrocytes into patients with Pelizaeus–Merzbacher disease.^7^ ³¹P MRS would be able to localize differences in the regional biochemistry of different areas of the brain after treatment was started. The increasing myelination, and concomitant increased synthesis of MPLs would alter the levels of MPL precursor and breakdown products; differences that would be observable with ³¹P MRS. This shows that *in vivo* ³¹P MRS has a role in characterizing the biochemical differences in Pelizaeus–Merzbacher disease; however, future studies are warranted to investigate whether ³¹P MRS could be used to monitor responses to treatment.

## Supplementary Material

fcac202_Supplementary_Data

## Data Availability

The ³¹P MRS and MRI data are available on request from the authors.

## Abbreviations

ACC =: medial anterior cingulate cortex
ADP =: adenosine diphosphate
ATP =: adenosine triphosphate
aWM =: anterior white matter
CK =: creatine kinase
dlPFC =: dorsolateral prefrontal cortex
GPC =: glycerophosphocholine
GPE =: glycerophosphoethanolamine
GM =: grey matter
HIP =: anterior/body of the hippocampus
iPL =: inferior parietal lobe
MPL =: membrane phospholipid
OCC =: occipital cortex
PC =: phosphocholine
PCC =: medial posterior cingulate cortex
PCr =: phosphocreatine
PCr + Cr =: phosphocreatine plus creatine
PE =: phosphoethanolamine
PLP1 =: proteolipid protein 1
³¹P MRS =: phosphorus magnetic resonance spectroscopy
pWM =: posterior white matter
sPL =: superior parietal lobe
STG =: superior temporal gyrus
STR =: striatum
THA =: thalamus
WM =: white matter
