# Supplementary material for: Altered high-energy phosphate and membrane metabolism in Pelizaeus–Merzbacher disease using phosphorus magnetic resonance spectroscopy
Source: Brain Commun. 2022 Aug 5;4(4):fcac202. doi: 10.1093/braincomms/fcac202 (PMC9396944; doi:10.1093/braincomms/fcac202)
Supplement: fcac202_Supplementary_Data [file fcac202_supplementary_data.pdf]

Supplementary Material

Supplementary Table 1: Mean metabolite levels (± SEM) of healthy controls and PMD patients for the 12 regions of interest.

			Voxel Regions											
			**aWM**	**pWM**	**dlPFC**	**sPL**	**iPL**	**ACC**	**PCC**	**STG**	**HIP**	**OCC**	**STR**	**THA**
**PE**	**HC**	Mean	7.85	7.32	7.93	7.63	7.48	7.85	6.74	7.79	8.01	7.84	7.41	6.90
		SEM	0.10	0.10	0.12	0.13	0.14	0.15	0.15	0.12	0.16	0.12	0.10	0.11
	**PMD**	Mean	7.85	7.45	7.93	7.84	7.56	8.18	7.09	7.44	7.33	7.75	7.40	6.48
		SEM	0.19	0.11	0.20	0.17	0.17	0.21	0.19	0.19	0.18	0.16	0.17	0.16
**PC**	**HC**	Mean	3.90	3.56	3.85	3.55	3.56	3.96	3.46	3.78	4.49	4.23	4.01	4.24
		SEM	0.09	0.09	0.10	0.09	0.10	0.11	0.14	0.09	0.11	0.12	0.08	0.08
	**PMD**	Mean	3.49	3.40	3.58	3.47	3.32	3.81	3.10	3.37	3.83	4.23	3.45	3.25
		SEM	0.23	0.09	0.16	0.13	0.12	0.34	0.18	0.16	0.15	0.11	0.16	0.12
**GPE**	**HC**	Mean	4.21	4.42	4.02	4.09	3.85	4.34	4.27	4.03	4.61	4.11	4.23	4.31
		SEM	0.08	0.09	0.09	0.12	0.09	0.13	0.15	0.11	0.10	0.13	0.09	0.10
	**PMD**	Mean	4.21	4.18	3.71	3.75	3.74	4.30	4.38	3.81	4.60	3.94	4.33	4.33
		SEM	0.17	0.12	0.15	0.15	0.13	0.31	0.20	0.14	0.16	0.13	0.14	0.17
**GPC**	**HC**	Mean	5.85	5.93	5.64	5.72	5.46	6.15	5.93	5.56	6.39	5.34	6.14	6.62
		SEM	0.10	0.09	0.11	0.17	0.13	0.20	0.17	0.13	0.15	0.16	0.11	0.12
	**PMD**	Mean	4.87	5.02	4.59	4.41	4.62	5.02	5.16	5.04	5.87	5.11	5.16	5.67
		SEM	0.19	0.15	0.26	0.16	0.12	0.35	0.23	0.15	0.19	0.33	0.19	0.17
**PCr**	**HC**	Mean	11.68	11.66	12.37	11.49	13.30	10.67	11.79	13.38	12.71	12.18	11.84	12.70
		SEM	0.18	0.10	0.23	0.17	0.27	0.26	0.17	0.25	0.26	0.18	0.17	0.14
	**PMD**	Mean	13.20	13.90	13.12	13.11	14.39	12.24	14.13	15.34	15.12	13.24	13.63	15.12
		SEM	0.28	0.16	0.44	0.28	0.43	0.52	0.25	0.52	0.51	0.17	0.32	0.25
**Pi**	**HC**	Mean	7.00	6.12	6.64	6.44	6.05	7.00	6.05	6.50	6.92	6.12	6.11	5.66
		SEM	0.42	0.18	0.36	0.39	0.28	0.83	0.29	0.29	0.40	0.32	0.27	0.07
	**PMD**	Mean	5.26	5.42	5.54	5.03	5.38	5.97	5.75	5.73	6.06	5.43	4.85	5.42
		SEM	0.30	0.11	0.41	0.23	0.27	0.74	0.44	0.30	0.42	0.28	0.27	0.11
**ATP**	**HC**	Mean	46.00	47.60	46.36	47.67	47.19	45.07	48.52	45.02	43.20	44.48	47.11	47.41
		SEM	0.49	0.37	0.42	0.45	0.35	0.70	0.61	0.40	0.50	0.43	0.34	0.30
	**PMD**	Mean	46.57	47.03	47.62	48.19	47.54	45.30	47.76	45.09	43.82	45.23	47.12	47.67
		SEM	0.72	0.38	0.62	0.49	0.48	1.24	0.69	0.44	0.86	0.36	0.48	0.46
**DN**	**HC**	Mean	3.83	3.74	3.70	3.77	3.66	4.21	3.85	4.00	3.87	4.16	3.82	3.62
		SEM	0.10	0.07	0.09	0.09	0.09	0.13	0.13	0.11	0.13	0.11	0.10	0.08
	**PMD**	Mean	4.54	3.67	4.24	3.86	3.56	4.87	3.57	4.10	3.77	4.00	4.25	3.74
		SEM	0.25	0.09	0.17	0.15	0.10	0.38	0.13	0.16	0.25	0.14	0.18	0.15
**MPL**	**HC**	Mean	9.67	9.67	9.48	9.64	9.44	10.75	9.38	9.95	9.80	11.53	9.32	8.53
		SEM	0.21	0.15	0.21	0.23	0.16	0.29	0.18	0.22	0.24	0.25	0.23	0.18
	**PMD**	Mean	10.00	9.93	9.68	10.34	9.90	10.32	9.05	10.08	9.61	11.07	9.82	8.31
		SEM	0.34	0.22	0.38	0.31	0.26	0.64	0.27	0.25	0.29	0.31	0.31	0.28

Supplementary Table 2: Mean spectral linewidth expressed as a full-width-at-half-maximum (± SEM) of the metabolites from healthy controls and PMD patients for the 12 regions of interest.

			Voxel Regions											
			**aWM**	**pWM**	**dlPFC**	**sPL**	**iPL**	**ACC**	**PCC**	**STG**	**HIP**	**OCC**	**STR**	**THA**
**PE**	**HC**	Mean	18.9	15.6	18.3	16.8	16.0	20.3	14.7	16.5	19.3	18.4	17.1	16.8
		SEM	0.4	0.4	0.4	0.6	0.5	0.6	0.7	0.4	0.9	0.4	0.3	0.5
	**PMD**	Mean	19.2	16.0	18.2	17.3	16.4	19.2	15.0	15.5	16.9	18.4	17.3	14.9
		SEM	1.9	0.5	0.5	0.6	0.7	2.6	0.9	0.7	1.0	0.8	0.8	0.6
**PC**	**HC**	Mean	18.9	15.6	18.3	16.8	16.0	20.3	14.7	16.5	19.3	18.4	17.1	16.8
		SEM	0.4	0.4	0.4	0.6	0.5	0.6	0.7	0.4	0.9	0.4	0.3	0.5
	**PMD**	Mean	19.2	16.0	18.2	17.3	16.4	19.2	15.0	15.5	16.9	18.4	17.3	14.9
		SEM	1.9	0.5	0.5	0.6	0.7	2.6	0.9	0.7	1.0	0.8	0.8	0.6
**GPE**	**HC**	Mean	18.3	15.5	18.2	17.9	16.3	19.4	14.9	17.3	16.2	19.2	15.0	13.4
		SEM	0.4	0.5	0.5	1.1	0.6	0.5	0.9	0.5	0.6	0.7	0.3	0.4
	**PMD**	Mean	19.5	16.3	18.9	16.8	16.4	18.9	16.1	17.7	16.9	18.8	16.5	13.9
		SEM	1.0	0.5	0.8	0.8	0.6	1.3	0.8	0.9	1.0	1.0	0.8	0.6
**GPC**	**HC**	Mean	18.3	15.5	18.2	17.9	16.3	19.4	14.9	17.3	16.2	19.2	15.0	13.4
		SEM	0.4	0.5	0.5	1.1	0.6	0.5	0.9	0.5	0.6	0.7	0.3	0.4
	**PMD**	Mean	19.5	16.3	18.9	16.8	16.4	18.9	16.1	17.7	16.9	18.8	16.5	13.9
		SEM	1.0	0.5	0.8	0.8	0.6	1.3	0.8	0.9	1.0	1.0	0.8	0.6
**PCr**	**HC**	Mean	12.2	8.1	12.2	10.3	9.8	12.3	8.1	9.3	8.2	9.6	8.5	7.1
		SEM	0.4	0.5	0.4	0.7	0.5	0.5	0.8	0.4	0.4	0.3	0.3	0.3
	**PMD**	Mean	12.6	9.7	12.6	11.1	9.8	10.3	9.6	10.1	8.8	10.1	9.6	8.2
		SEM	1.2	0.5	0.5	0.7	0.4	0.8	0.7	0.5	0.5	0.6	0.4	0.4
**Pi**	**HC**	Mean	27.1	17.9	23.0	25.0	21.1	37.0	19.0	21.0	25.1	23.2	21.7	14.9
		SEM	3.0	1.3	2.4	3.1	2.2	5.7	2.7	2.0	3.1	2.5	2.2	0.3
	**PMD**	Mean	19.3	15.8	20.6	19.9	17.7	23.3	18.0	18.7	22.2	18.1	18.9	15.0
		SEM	2.3	0.6	2.6	2.2	1.9	5.5	3.2	2.9	3.4	2.1	2.3	0.6
**ATP**	**HC**	Mean	17.0	13.2	16.1	15.4	14.1	17.7	13.2	13.3	14.5	15.0	14.2	11.7
		SEM	0.7	0.6	0.6	0.8	0.7	0.9	0.9	0.4	1.1	0.6	0.7	0.4
	**PMD**	Mean	19.7	14.2	21.5	17.7	16.1	23.3	13.9	14.7	16.1	16.8	15.7	12.9
		SEM	2.0	0.6	2.0	1.2	1.1	4.6	0.9	0.8	1.9	1.2	1.2	0.8
**DN**	**HC**	Mean	17.2	13.3	16.3	15.5	14.4	18.5	13.4	13.4	14.8	15.1	14.0	11.7
		SEM	0.8	0.6	0.6	0.9	0.7	1.1	1.0	0.5	1.2	0.7	0.8	0.4
	**PMD**	Mean	20.2	14.2	22.0	18.2	16.7	24.1	13.9	15.0	17.0	17.3	15.9	13.3
		SEM	2.2	0.7	2.1	1.4	1.3	4.7	0.9	0.9	2.4	1.3	1.3	1.0
**MPL**	**HC**	Mean	43.6	43.3	42.5	43.6	43.5	45.5	44.0	43.0	43.2	44.8	42.7	42.1
		SEM	0.6	0.6	0.6	0.7	0.7	0.8	0.5	0.9	1.2	0.8	1.1	0.7
	**PMD**	Mean	44.6	42.2	45.0	45.0	43.2	43.9	41.0	41.0	42.9	41.4	42.8	39.7
		SEM	1.2	0.6	1.2	1.1	1.1	2.1	1.0	1.0	1.2	0.7	1.0	0.7

Supplementary Table 3: Results from the main group effect and group by region interaction for the metabolite ratios relative to ATP.

Metabolite Ratio	Main Group Effect	Main Group by Region Interaction Effect
**PE/ATP**	n.s. (χ^2= 0.46; p= 0.50)	n.s. (χ^2= 9.14; p= 0.61)
**PC/ATP**	**↓ PMD vs Controls (χ^2= 8.42; p= 0.0037)**	n.s. (χ^2= 12.86; p= 0.30)
**GPE/ATP**	n.s. (χ^2=1.85; p= 0.17)	n.s. (χ^2= 9.12; p= 0.61)
**GPC/ATP**	**↓ PMD vs Controls (χ^2= 16.00; p< 0.0001)**	n.s. (χ^2= 11.23; p= 0.42)
**PCr/ATP**	**↑ PMD vs Controls (χ^2= 13.20; p= 0.0003)**	**χ^2= 23.12; p= 0.017 Post-hoc analyses of regions demonstrating significant increased PCr/ATP in PMD vs Controls: pWM, sPL, PCC, HIP, STR and THA (all p< .0042)**
**Pi/ATP**	**↓ PMD vs Controls (χ^2= 12.39; p= 0.0004)**	n.s. (χ^2= 12.37; p= 0.34)

Supplementary Table 4: Mean metabolite ratios relative to ATP (± SEM) of healthy controls and PMD patients for the 12 regions of interest.

			Voxel Regions											
			**aWM**	**pWM**	**dlPFC**	**sPL**	**iPL**	**ACC**	**PCC**	**STG**	**HIP**	**OCC**	**STR**	**THA**
**PE/ ATP**	**HC**	Mean	0.172	0.154	0.172	0.161	0.159	0.175	0.140	0.174	0.187	0.177	0.158	0.146
		SEM	0.003	0.003	0.003	0.003	0.004	0.005	0.004	0.003	0.006	0.004	0.003	0.003
	**PMD**	Mean	0.170	0.159	0.167	0.163	0.159	0.182	0.149	0.165	0.169	0.172	0.158	0.136
		SEM	0.005	0.003	0.005	0.004	0.004	0.007	0.005	0.004	0.006	0.004	0.004	0.004
**PC/ ATP**	**HC**	Mean	0.086	0.075	0.084	0.075	0.076	0.089	0.072	0.084	0.105	0.096	0.085	0.090
		SEM	0.002	0.002	0.003	0.002	0.002	0.003	0.004	0.002	0.003	0.003	0.002	0.002
	**PMD**	Mean	0.076	0.073	0.076	0.072	0.070	0.085	0.065	0.075	0.089	0.094	0.074	0.069
		SEM	0.006	0.002	0.004	0.003	0.003	0.008	0.004	0.004	0.004	0.003	0.004	0.003
**GPE/ ATP**	**HC**	Mean	0.092	0.093	0.087	0.086	0.082	0.097	0.089	0.090	0.108	0.093	0.090	0.091
		SEM	0.002	0.002	0.002	0.003	0.002	0.003	0.004	0.003	0.003	0.003	0.002	0.002
	**PMD**	Mean	0.091	0.089	0.079	0.078	0.079	0.097	0.093	0.085	0.107	0.088	0.092	0.092
		SEM	0.004	0.003	0.004	0.004	0.003	0.009	0.006	0.003	0.005	0.004	0.003	0.004
**GPC/ ATP**	**HC**	Mean	0.128	0.125	0.123	0.121	0.117	0.137	0.123	0.124	0.150	0.121	0.131	0.140
		SEM	0.003	0.003	0.003	0.004	0.003	0.005	0.004	0.003	0.005	0.004	0.003	0.003
	**PMD**	Mean	0.105	0.107	0.097	0.092	0.098	0.113	0.109	0.112	0.136	0.114	0.110	0.120
		SEM	0.005	0.004	0.006	0.004	0.003	0.010	0.006	0.004	0.006	0.008	0.004	0.004
**PCr/ ATP**	**HC**	Mean	0.255	0.246	0.268	0.242	0.282	0.238	0.244	0.298	0.295	0.275	0.252	0.269
		SEM	0.004	0.003	0.006	0.004	0.006	0.006	0.005	0.007	0.006	0.005	0.004	0.004
	**PMD**	Mean	0.285	0.296	0.277	0.273	0.304	0.272	0.297	0.343	0.350	0.293	0.290	0.318
		SEM	0.009	0.005	0.011	0.007	0.011	0.015	0.007	0.014	0.014	0.004	0.007	0.006
**Pi/ ATP**	**HC**	Mean	0.157	0.130	0.146	0.138	0.130	0.162	0.126	0.147	0.166	0.139	0.131	0.120
		SEM	0.011	0.005	0.010	0.010	0.007	0.021	0.008	0.008	0.012	0.008	0.007	0.002
	**PMD**	Mean	0.115	0.116	0.118	0.105	0.114	0.136	0.122	0.128	0.141	0.121	0.104	0.114
		SEM	0.008	0.003	0.011	0.005	0.007	0.022	0.011	0.008	0.012	0.008	0.006	0.003
